# Antisynthetase Syndrome With Predominant Pulmonary Involvement: A Case Report

**DOI:** 10.7759/cureus.43966

**Published:** 2023-08-23

**Authors:** Beatriz Lima Corrêa de Araújo, David R Victor, Heloísa Maria Farias Fontes, Rayana Maria Caminha Mendes Gomes, Leonardo Lima Corrêa de Araújo

**Affiliations:** 1 Department of Internal Medicine, Hospital Barão de Lucena, Recife, BRA; 2 Medical School, Universidade de Pernambuco, Recife, BRA; 3 Medical School, Universidade Federal de Pernambuco, Recife, BRA

**Keywords:** anti-jo1, myositis, rituximab, interstitial lung disease, antisynthetase syndrome

## Abstract

Antisynthetase syndrome (ASyS) is an autoimmune disease characterized by the presence of aminoacyl-transfer RNA synthetase antibodies. Its clinical presentation is variable and may include interstitial lung disease (ILD), myositis, arthritis, fever, Raynaud’s phenomenon, and “mechanic’s hands.” ILD is more prevalent in this entity when compared to other idiopathic inflammatory myopathies and imparts greater severity to the condition. Here, we report the case of a 42-year-old female patient who sought care for severe ILD and persistent fever. Her diagnosis was made only after the detection of anti-Jo1 autoantibodies. Treatment was refractory to both prednisone monotherapy and cyclophosphamide pulse therapy, requiring the introduction of rituximab. A high degree of clinical suspicion is required to allow early diagnosis of ASyS in patients with pulmonary involvement in the absence of accompanying muscle weakness or other clinical symptoms.

## Introduction

Antisythetase syndrome (ASyS) is a rare systemic autoimmune disease classified as one of the idiopathic inflammatory myopathies (IIMs) [1,2]. ASyS is characterized by the development of specific antisynthetase autoantibodies (ASAs) each targeting unique aminoacyl transfer RNA (tRNA) synthetases [1,2]. These enzymes are involved in protein synthesis, as they are responsible for connecting amino acid molecules to their specific tRNA counterparts [2]. Among ASAs, the most common is anti-Jo1, which targets the enzyme histidyl-tRNA synthetase [1,2].

Regarding its presentation, ASyS is characterized by a classic triad of arthritis, myositis, and interstitial lung disease (ILD) [3]. Additional findings such as Raynaud’s phenomenon, unexplained fever, and mechanic’s hands can also be observed [3]. However, it is important to note that not all ASyS patients exhibit the complete triad of manifestations. A retrospective analysis by Cavanga et al. included 225 patients diagnosed with ASyS, all of whom tested positive for the anti-Jo1 autoantibody [3]. Interestingly, only 19.5% of these patients (44 patients) initially presented with the complete triad [3]. However, at the end of the follow-up, the number of patients with the complete triad had increased to 50% (113 patients) due to disease progression [3].

In the same study, within the subgroup of patients with myositis, some individuals showed no clinical muscle weakness either at disease onset or at the end of the follow-up [3]. Instead, they presented solely with laboratory or instrumental signs of muscle involvement [3]. A case report of ASyS without concurrent clinical muscle weakness was also published in the literature [4].

Due to its rarity, ASyS diagnosis is not usually suspected during clinical practice. Furthermore, when ASyS presents without clinical signs of myositis and arthritis, achieving a diagnosis becomes even more difficult. Therefore, in publishing this case report, we aim to bring attention to a challenging presentation of a rare disease.

## Case presentation

A 42-year-old woman was admitted to a hospital with a clinical condition that had persisted for four years. Throughout this period, she experienced progressive dyspnea, persistent intermittent fever, and unmeasured weight loss. Four months before her hospital admission, her respiratory pattern had deteriorated considerably, necessitating home oxygen therapy.

During her initial physical examination, the patient was found to be febrile, dyspneic, and tachypneic, requiring oxygen support through a nasal catheter at a flow of 5 L/minute. Her pulmonary auscultation revealed fine rales in both lung bases. There was no palpable lymph node enlargement, digital clubbing, dactylitis, arthritis, joint deformities, muscle weakness, or skin/mucosal lesions.

Initial laboratory tests (Table 1) revealed microcytic and hypochromic anemia, a normal differential white blood cell count, thrombocytosis, hypoalbuminemia, and an elevated C-reactive protein level. Viral serologies for hepatitis B, hepatitis C, syphilis, and HIV were negative. Arterial blood gas analysis (Table 2) indicated hypoxemia, with a partial pressure of oxygen (PaO_2_) of 49 mmHg. A reverse transcription-polymerase chain reaction test for SARS-CoV-2 yielded a negative result.

**Table 1:** Initial blood biochemical assessment and viral serologies requested for the patient’s evaluation.

Parameter	Result	Reference range
Hemoglobin	10.3 g/dL	12–16 g/dL
Hematocrit	30.3%	35–47%
Mean corpuscular volume	79 fL	80.0–100 fL
Mean corpuscular hemoglobin	20 pg	27–32 pg
White blood cell count	9,370/mm^3^	4,000–11,000/mm^3^
Platelets	509,000/mm^3^	150,000–450,000/mm^3^
Urea	16 mg/dL	13–43 mg/dL
Creatinine	0.6 mg/dL	0.60–1.2 mg/dL
Albumin	2.62 g/dL	3.5–5 g/dL
Thyroid-stimulating hormone	3.69 mU/L	0.3–4.0 mU/L
Aspartate transferase	53 U/L	5–40 U/L
Alanine aminotransferase	54 U/L	7–56 U/L
Alkaline phosphatase	77 U/L	46–120 U/L
Gamma-glutamyl transferase	78 U/L	7–45 U/L
Total bilirubin	0.6 mg/dL	0.1–1.2 mg/dL
Lactate dehydrogenase	467 U/L	120–246 U/L
Sodium	138 mmol/L	135–145 mmol/L
Potassium	3.3 mmol/L	3.5–5.5 mmol/L
C-reactive protein	6.7 mg/dL	0.3–1 mg/dL
Free thyroxine	1.69 ng/dL	0.7–1.8 ng/dL
Hepatitis B surface antigen	Nonreactive	
Hepatitis B surface antibody	Nonreactive	
Hepatitis B core antibody IgM	Nonreactive	
Hepatitis B core antibody IgG	Nonreactive	
Hepatitis C antibody	Nonreactive	
Human immunodeficiency virus 1 and 2 antibody	Nonreactive	
Treponemal antibody test	Nonreactive	

**Table 2:** Initial arterial blood gas analysis requested for the patient’s evaluation. The exam was performed while the patient was breathing ambient air.

Parameter	Result	Reference range
pH	7.48	7.35–7.45
Partial pressure of oxygen	49 mmHg	75–100 mmHg
Partial pressure of carbon dioxide	37 mmHg	35–45 mmHg
Blood oxygen saturation	89%	94–100%

**Figure 1:**
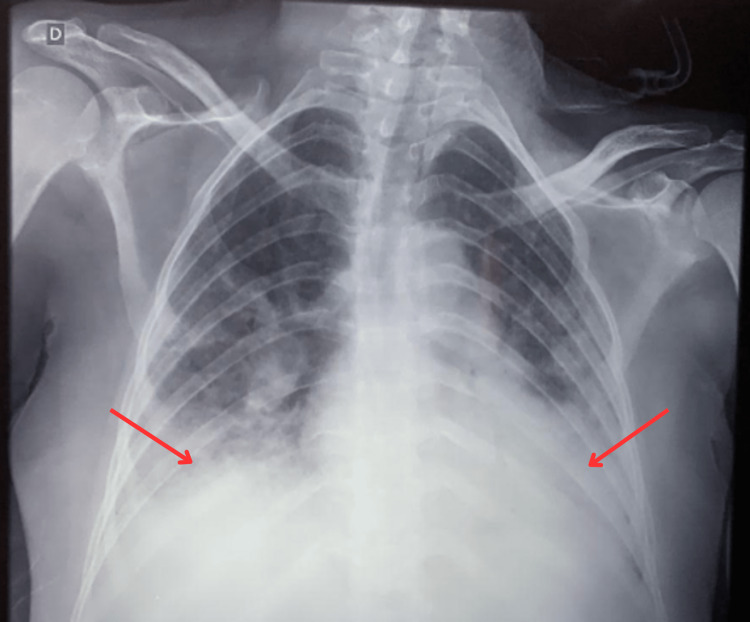
Initial chest radiography registered in the anteroposterior view showing basal consolidative opacities in both lungs, as indicated by the red arrows.

The initial chest radiography (Figure 1) revealed predominantly basal consolidative opacities in both lungs, with an increased left hilum and preserved heart area. There was no evidence of pleural effusion.

Antibiotic therapy consisting of ceftriaxone and azithromycin was introduced in the suspicion of community-acquired pneumonia superimposed on pre-existing ILD. Later, because there was no apparent clinical improvement, the antimicrobial treatment was altered to cefepime, and oxygen therapy was optimized by gradually increasing the oxygen supply.

**Figure 2:**
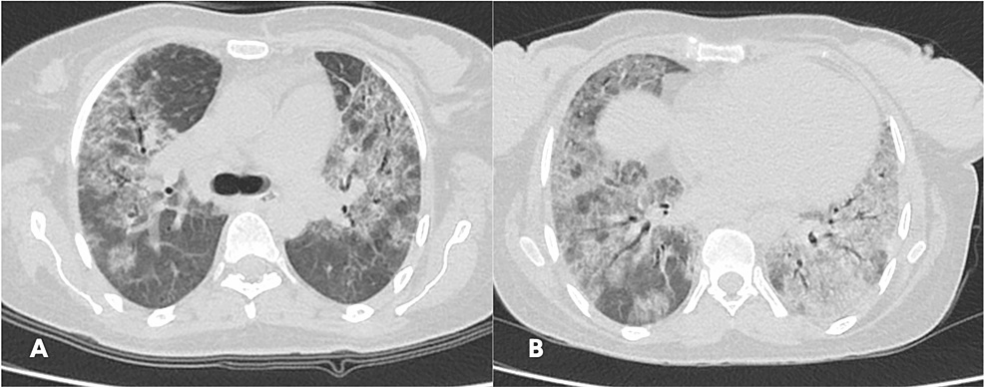
Contrast-enhanced CT scan demonstrating ground-glass opacities and diffuse septal thickening (A, B) with a basal predominance (B).

A contrast-enhanced CT found diffuse ground-glass opacities with septal thickening (Figures 2A, 2B) and a basal predominance (Figure 2B), characterizing a mosaic attenuation pattern. Additionally, there were multiple enlarged lymph nodes in the mediastinum and hilar regions, with the largest cluster located in the subcarinal region, measuring 5.3 × 2.8 × 2.2 cm. No signs of honeycombing or effusions were present. The dimensions of the heart chambers were normal.

Subsequently, the result of a bronchoalveolar lavage performed before hospitalization was retrieved. The examination revealed a predominance of lymphocytes, with negative results for both mycobacteria and fungi.

The investigation continued with an autoantibody panel request, which revealed an antinuclear antibody titer of 1:320 with a mix of cytoplasmic fine-speckled and nuclear-speckled patterns, anti-Ro levels of >240 IU/mL (reference value of <7 IU/mL), and rheumatoid factor levels of 121.4 IU/mL (reference value of <20 IU/mL). Anti-La, anti-Sm, perinuclear antineutrophil cytoplasmic antibodies, cytoplasmic antineutrophil cytoplasmic antibodies, and anti-Scl70 were all negative.

In view of these findings, corticosteroid therapy with prednisone at a dosage of 1 mg/kg/day was initiated. However, after two weeks of treatment, the patient maintained an uncomfortable breathing pattern and still required continuous oxygen supplementation at the same flow rate. Therefore, the investigation proceeded with lung and lymph node biopsies to rule out lymphoma as a potential differential diagnosis. The histopathological analysis revealed non-specific fibrotic interstitial pneumonitis and reactive inflammation in the lymph node sample. There was no evidence of granulomas, neoplastic cells, or viral inclusions.

Afterward, an anti-Jo1 antibody screening test, which had been requested alongside the previous antibody panel, was retrieved. The positive result led to the diagnosis of ASyS. Hence, the patient’s oral corticosteroid treatment was continued, and a monthly pulse therapy of cyclophosphamide at a dosage of 750 mg/m^2^ was initiated for six months. Moreover, due to her corticosteroid usage, prophylaxis for *Pneumocystis jirovecii* with trimethoprim-sulfamethoxazole was started. Ten days after the first cycle, the patient was successfully withdrawn from oxygen support and has since remained without any further respiratory assistance.

**Figure 3:**
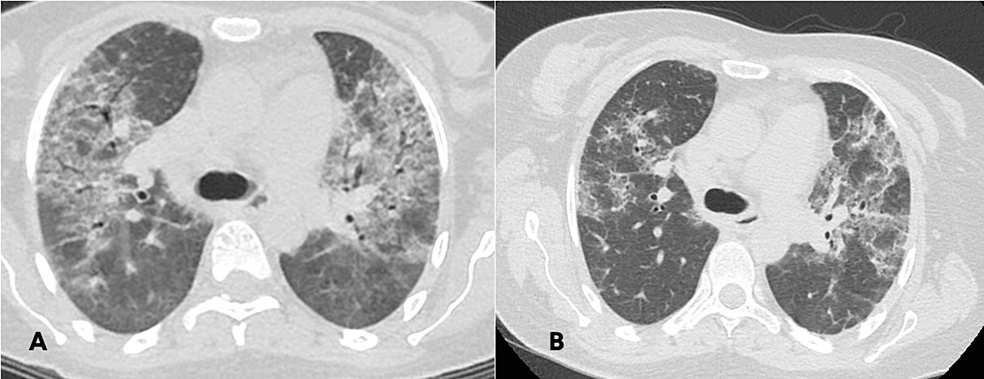
Comparison of tomographic images at the level of the tracheal carina before (A) and after (B) the therapeutic intervention. Images show partial resolution of tomographic findings.

Following the completion of treatment, although there was considerable clinical improvement, the tomographic findings were only partially resolved (Figures 3A, 3B), and there was still evidence of a restrictive ventilatory disorder according to two different spirometry results (Table 3). Therefore, due to the enduring nature of her condition, it was determined that the treatment would be continued with rituximab for refractory ASyS. The prescribed dosage was 1 g administered at a two-week interval, with another dose after six months.

**Table 3:** Results of the first spirometry performed while under immunosuppression with cyclophosphamide. Subsequently, a second spirometry was performed after the completion of the regimen. Despite the conclusion of the treatment, the severe restrictive pattern persists. Additionally, there was no response to the administration of inhaled bronchodilators. FVC: forced vital capacity; FEV1: forced expiratory volume in one second

	First spirometry	Second spirometry
FVC	1.41 (41%)	1.30 (38%)
FEV1	1.07 (38%)	0.92 (33%)
FEV1/FVC	75.9%	70.8%

## Discussion

As each aminoacyl tRNA synthetase is responsible for attaching one of the 20 essential amino acids to its corresponding tRNA molecule, it was assumed that 20 ASAs would exist [5]. However, so far, only 11 have been identified [6]. In general, patients present with a single ASA [2], although cases of coexistence have been reported in the literature [7]. Despite these variations in autoantibody profiles, data from the American and European Network of Antisynthetase Syndrome demonstrated that ASyS exhibits broadly similar clinical manifestations, disease course, and survival rates [8].

An additional relevant antibody found in our patient was anti-Ro/SSA. A study by La Corta et al. found a higher prevalence of anti-RO/SSA in patients with ASyS when compared to patients with classic polymyositis/dermatomyositis [9]. The same study showed that the association holds clinical significance, as the subgroup of ASyS patients who tested positive for anti-RO/SSA developed more severe ILD [9]. This correlation may have also played a role in our patient’s ILD severity. Lastly, although the detection of anti-RO/SSA antibodies may prompt consideration of Sjögren’s syndrome, as they are found in 70% of patients with Sjögren’s syndrome [10], it is worth noting that our patient did not exhibit any clinical sicca symptoms.

Currently, there are two criteria for ASyS diagnosis [1,2]. The first, introduced by Connor et al. in 2010, required a positive serological test for an ASA in association with at least one of the following conditions: evidence of myositis according to the Bohan and Peter criteria; evidence of ILD; evidence of arthritis; persistent unexplained fever; the presence of Raynaud’s phenomenon; or the presence of mechanic hands (Table 4) [11]. Afterward, in 2011, Solomon et al. proposed other alternative stricter criteria (Table 4) [12]. Until today, definitions of ASyS vary in the literature, with no validated diagnostic criteria [13]. Our case was diagnosed in accordance with Connor et al.’s criteria as our patient had a positive serological test for the anti-Jo1 antibody associated with ILD and unexplained persistent fever.

**Table 4:** Proposed criteria for the diagnosis of ASyS. ASA: antisynthetase autoantibody; ILD: interstitial lung disease

	Connors et al. (2010) [11]	Solomon et al. (2011) [12]
Serology	Required: Positive serologic testing for an ASA	Required: Positive serologic testing for an ASA
Clinical features	Plus one more of the following: Myositis by the Bohan and Peter criteria; ILD; evidence of arthritis by clinical examination, radiographic findings, or patient self-report; unexplained, persistent fever; Raynaud’s phenomenon; mechanic’s hands	Plus two major or one major and two minor criteria. Major: ILD; myositis by the Bohan and Peter criteria. Minor: arthritis; Raynaud’s phenomenon; mechanic’s hands

Furthermore, there may be an interposition of the diagnosis of ASyS with that of interstitial pneumonia with autoimmune feature (IPAF) [14]. This new entity was proposed in 2015 by a combined task force of the European Respiratory Society and the American Thoracic Society [14,15]. For its diagnosis, it is necessary to fulfill two of three previously stipulated domains [15]. However, if the patient meets the criteria for other connective diseases, the diagnosis of IPAF would be excluded [15]. Nevertheless, due to the absence of definitive criteria for ASyS, it is possible that a patient does not fulfill Solomon et al.’s criteria while satisfying Connor et al.’s criteria [14]. Thus, the patient could be diagnosed with either ASyS or IPAF [14]. This phenomenon is in line with the case of our patient.

With regards to ASyS treatment, it should be based on the patient’s clinical manifestations, focusing on its severity and the organs involved [2]. Because ILD has been recognized as a leading contributor to ASyS morbidity and mortality [1,2], it often dictates therapeutics [2]. As there are no FDA-approved drugs, most regimens are based on retrospective studies and expert opinion [2], drawing from data from previous studies on dermatomyositis and polymyositis [1].

Thus, the first-line treatment for ILD associated with myositis involves corticosteroid therapy with oral prednisone 1 mg/kg/day or pulse therapy with methylprednisolone 1,000 mg for three days [2,16]. Nonetheless, more recent research has shown that the up-front association of steroids and immunosuppressive drugs can be more effective than corticoid monotherapy [17,18]. Among these immunosuppressive medications are azathioprine, mycophenolate mofetil, tacrolimus, rituximab, and cyclophosphamide [1]. In this case, treatment was started with prednisone 1 mg/kg/day, but, later on, cyclophosphamide and rituximab were utilized.

As it relates to the use of cyclophosphamide, it is generally administered orally or intravenously in acute or refractory ILD cases at a dose of 300-800 mg/m^2^ every four weeks [16]. This reservation of cyclophosphamide prescription is due to its notable toxicity [1,2], which can lead to malignancies, opportunistic infections, heart failure, and sterility [1]. Regarding the risk of sterility, a study by Dimitrios et al. demonstrated that cyclophosphamide pulse therapy can lead to sustained amenorrhea [19]. This risk is more prominent in older patients or those undergoing longer treatments [19]. Given that our patient had already borne offspring and expressed no intention of conceiving again, the initiation of cyclophosphamide was deemed necessary due to the severity of her clinical condition, which was also refractory to prednisone monotherapy. Fortunately, she had no complications after starting the medication.

Rituximab has also been previously employed with success as a treatment for severe refractory ASyS [20]. Studies have demonstrated that its use in ASyS-associated ILD leads to improvements in the symptomatology, pulmonary function test, and radiological profile [2]. Hence, a retrospective cohort study by Andersson et al., which analyzed 24 patients with severe ASyS treated with rituximab, showed a 24% improvement in the predicted forced vital capacity, a 22% improvement in the predicted forced expiratory volume in one second, a 17% improvement in the predicted diffusing capacity of the lungs for carbon monoxide, and a mean reduction in lung parenchyma affected by ILD in CT scans of 17% [21]. Our patient was started on rituximab as she did not respond to cyclophosphamide.

In addition to treatment, different authors have brought attention to the importance of starting prophylaxis for *Pneumocystis jirovecii* in ASyS patients, especially those treated with immunosuppressive therapy [1,2,17]. This attention should be greater in patients treated with a more aggressive immunosuppressive regimen or those treated with a combination of different medications [2]. For this reason, we prescribed trimethoprim-sulfamethoxazole as a prophylactic agent. It is also worth mentioning Andersson et al. finding in their rituximab study that all patients who developed infection-related serious adverse events (half being by *Pneumocystis jirovecii*) were treated with cyclophosphamide before or directly after the first rituximab cycle [21]. As this treatment route was also implemented in our patient, prophylaxis was maintained during rituximab use.

## Conclusions

ASyS is a rare disease, and its occurrence without concurrent arthritis or clinical muscle weakness renders the diagnostic process even more challenging. Thus, this case report emphasizes the importance of clinicians considering ASyS as a possible differential diagnosis in patients with idiopathic ILD. When faced with such cases, serological evaluation of the main ASAs emerges as an important diagnostic tool. Finally, further studies are required to establish definitive diagnostic criteria and compare various therapeutic options for treating the disease.
